# Cholera

**Published:** 1849-05

**Authors:** 


### CHOLERA.

From all points in this country we receive intelligence of the decline of cholera. At New Orleans it ceases to excite any uneasiness, although deaths from it continue to be reported. No mention is made of the disease in the last Nashville papers. In St. Louis fatal cases were occasionally occurring at the last dates, and boats from that city, as well as from New Orleans, have brought cholera patients to Louisville within a few days. In this place scattering cases are spoken of by our physicians, but there does not appear to be the slightest tendency in the disease to become general, and many even doubt whether we have yet had a single indigenous case of cholera. We are not of that number. The last European intelligence is that the epidemic was committing fearful ravages among the French troops stationed at Paris.

